# Pressure build-up and stress variations within the Earth’s crust in the light of analogue models

**DOI:** 10.1038/s41598-018-38256-1

**Published:** 2019-02-19

**Authors:** Evangelos Moulas, Dimitrios Sokoutis, Ernst Willingshofer

**Affiliations:** 1 Institute of Earth Sciences, University of Lausanne, Lausanne, Switzerland; 2 Department of Earth Sciences, Utrecht University, Utrecht, Netherlands; 3 University of Oslo, Department of Geosciences, PO Box 1047 Blindern, NO-316 Oslo, Norway

## Abstract

Strength contrasts and spatial variations in rheology are likely to produce significant stress differences in the Εarth’s crust. The buildup and the relaxation of stresses have important consequences for the state of stress of the brittle crust, its deformational behaviour and seismicity. We performed scaled analogue experiments of a classic wedge-type geometry wherein we introduced a weak, fluid-filled body representing a low-stress heterogeneity. The experiments were coupled to direct pressure measurements that revealed significant pressure differences from their surrounding stressed matrix. The magnitude of the pressure variations is similar to the magnitude of the differential stress of the strongest lithology in the system. When rocks with negligible differential stresses are considered, their pressure can be more than twice larger than the surrounding lithostatic stress. The values of the pressure variations are consistent with the stresses that are estimated in analytical studies. This behaviour is not restricted to a particular scale or rheology, but it requires materials that are able to support different levels of stress upon deformation. For non-creeping rheological behaviours, the stress and pressure variations are maintained even after deformation ceases, implying that these stress variations can be preserved in nature over geological timescales.

## Introduction

The state of stress in the Earth’s upper crust is important for a variety of geodynamic phenomena such as earthquakes, volcanic eruptions and the strain evolution of rocks and regions. Some of the first mechanical investigations considering the Earth’s crust were based on the study of simple materials that can be used as scaled analogues in space and time^1–3^. Such models can provide useful insights especially on the evolution of complex strain patterns in deforming regions of the crust and the lithosphere in general^4–6^. The study of crustal wedges^7^ and the mechanics of thin-skinned tectonics^8–11^ are examples of such mechanical investigations whose applicability lies beyond the conditions of the shallow upper crust^12,13^. Over the past years many studies, experimental or theoretical, have reported the effects of initial geological configuration^14^, rheology^15,16^, surface processes and sediment distribution^17^ on the mechanics and the evolution of accretionary complexes and fold and thrust belts^18–20^. In addition, recent investigations suggest that the consideration of the heterogeneous distribution of field variables, like porosity and pore-pressure ratio, may result in significant deviations from the classic wedge solutions^21^. Apart from the aforementioned heterogeneities, the effect of heterogeneous stress distribution in the Earth’s crust also remains largely unexplored. The influence of strength heterogeneities in the overall mechanics of the crust is difficult to be constrained due to the limited stress measurements from large depths and the heterogeneous nature of stress distribution.

Heterogeneous stress and mean stress (pressure) distributions significantly influence the mechanical state of the crust because they affect the style of deformation and exert first-order controls on fluid migration. In this study, we develop a novel, yet simple method for investigating the stress evolution in scaled analogue experiments of heterogeneous brittle wedges. We introduced a fluid-filled inclusion within the brittle layer where we could monitor the hydrostatic pressure during plastic deformation. Furthermore, we present the results of experiments with direct pressure measurements to show that strength heterogeneities, such as weak bodies embedded in a stressed brittle layer can experience significant (>100%) pressure variations from the initial lithostatic stress.

Our results are consistent with classical mechanical views of wedge mechanics and reveal that the accurate knowledge of the rocks strength and their distribution is essential for the understanding of the dynamics and processes that occur in tectonically stressed regions. Finally, our measurements show that a significant part of these stress perturbations and non-lithostatic pressures are preserved even after the deformation stops and even if the wedge material has reached its yield point. The aim of this study is to show that stress variations that can occur in the crust is controlled not only from the maximum strength of rocks but also from the geometrical configuration and the far-field stress conditions as envisaged by analogue experiments, simple mechanical solutions and thermomechanical modelling.

## Results

**Figure 1:**
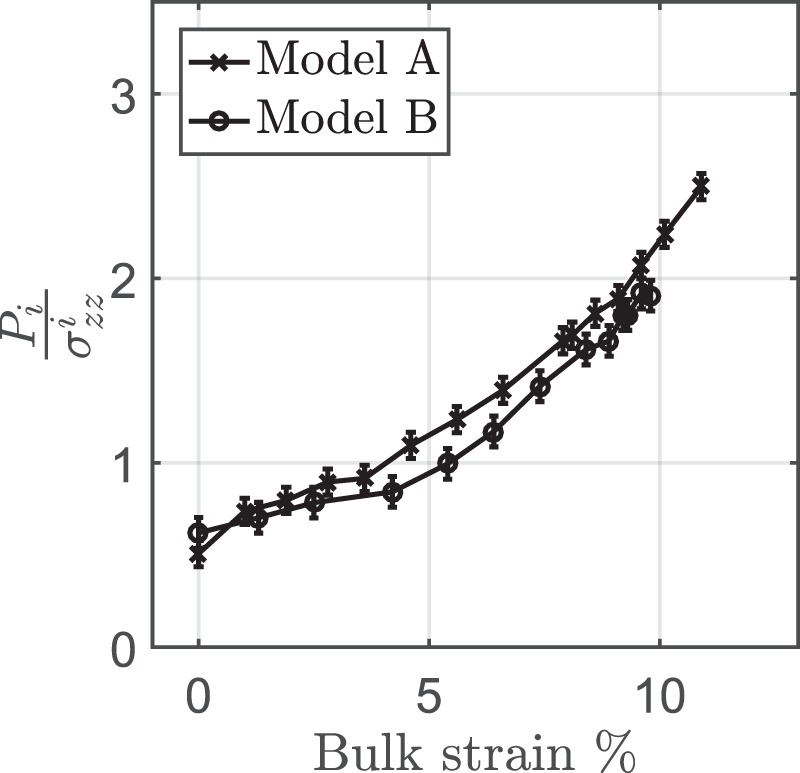
Inclusion pressure evolution during the deformation experiments (Models A, B). Bulk strain has been calculated as the displacement of basal slip over the initial length of the model (1 m). The inclusion (*P*_*i*_) pressure is normalized over the initial lithostatic stress (${\sigma }_{zz}^{i}$). Both experiments were stopped at relative low bulk strains (<12%).

We used an experimental configuration that is typical for brittle accretionary wedges^22^ (see METHODS for further details). In these experiments, a brittle layer is pulled from below against a rigid backstop thus creating a thrust belt. A water-filled inclusion was placed in the bottom of the brittle layer in the center of the model. The inclusion was connected to a small rubber tube that was open to the surface and allowed the monitoring of the inclusion pressure during the deformation experiments. We performed two deformation experiments that had very similar configurations. The results show that with progressing deformation several thrusts develop during thickening. The thrusts are propagating towards the thin (forward) part of the wedge and, to a first approximation they are similar with classic wedge models^22^. The inclusion pressure (*P*_*i*_) is increasing with increasing deformation and it significantly deviates from the initial lithostatic stress (${\sigma }_{zz}^{i}$) (Fig. 1). The initial lithostatic stress is used as a reference constant value to scale our results. The final value of the inclusion pressure *P*_*i*_ reaches up to 2.0-2.5 times higher than the initial lithostatic stress ${\sigma }_{zz}^{i}$.

**Figure 2:**
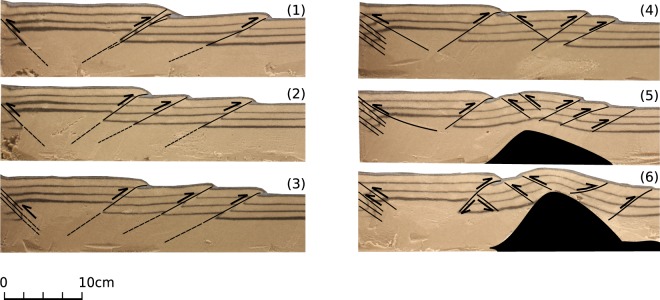
Cross sections of Model A after the end of the experiment. The maximum topography increase on top of the weak inclusion is 40% with respect to the initial layer thickness. Numbers 1–6 indicate sections from the boundary towards the central axis of the model. The weak inclusion is indicated by black color (sections 5,6).

The experiments were stopped at relative low bulk strain (<12% bulk horizontal strain). At the end of the experiments, the deformation rate was zero and *P*_*i*_ was measured. The pressure relaxation was negligible. After deformation, the model was soaked in water, and cut longitudinally to expose cross sections for photographs of the internal structures (Fig. 2). The final sections show typical shear bands (thrust faults) that dip towards the hinterland and a few antithetic ones (Fig. 2, profiles 1–3). Towards the center of the model, the deformation of the weak inclusion created a pop-up structure with small antithetic shear bands that root at the weak inclusion (Fig. 2, profiles 4-6). The relative increase in topography above the inclusion is 40% of the initial layer thickness (*δH* = 0.4 *L*) and this is mostly due to the deformation of the inclusion. The change in sand thickness just above the inclusion is negligible as demonstrated by the black marker lines (Fig. 2, profile 6).

The topography increase above the inclusion occurs as a response to the inclusion deformation. If the inclusion was not confined, the increase on the hydrostatic head must equal to the height increase of the hydrostatic column. For a height increase equal to *δH*, the corresponding hydrostatic pressure increase would be:

1 $$\delta {P}_{i}={\rho }_{f}g\delta H$$

where *ρ*_*f*_ is the fluid density and *g* is the acceleration of gravity. The initial lithostatic pressure (${\sigma }_{zz}^{i}$) is given by:

2 $${\sigma }_{zz}^{i}={\rho }_{s}gL$$

where *ρ*_*s*_ is the density of the quartz sand and therefore the normalized hydrostatic pressure increase would be equal to:

3 $$\frac{\delta {P}_{i}}{{\sigma }_{zz}^{i}}=\frac{{\rho }_{f}}{{\rho }_{s}}\frac{\delta H}{L}\approx 0.27$$

Based on Fig. 1, the normalized hydrostatic pressure increase was between 1.4 and 1.9 and thus the pressure of the inclusion increased mostly due to the applied tractions on its surface.

## Discussion

The increase of inclusion pressure suggests that the stress in that region is not dominated by the lithostatic load but it has been affected by tectonic stresses. The thickness variation that developed in the inclusion is not sufficient to explain the large pressure increase within the inclusion (Fig. 2). Simple analytical solutions suggest that pressure, expressed as a mean stress, is expected to vary in highly stressed regions of the crust^23,24^. Weak bodies in the vicinity of stressed rocks have nearly isotropic state of stress and because of their low differential stress their pressure need not be lithostatic^25,26^. Although not intuitive, this result is in perfect agreement with classic continuum mechanics and it is a requirement of force balance.

**Figure 3:**
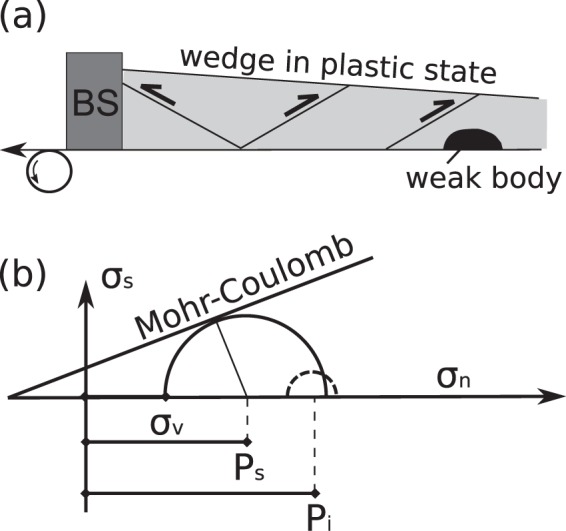
(**a**) Schematic diagram that shows a wedge in plastic state (not to scale). (**b**) A solid Mohr circle indicates the state of stress at a point adjacent to the weak body. Shear stresses are indicated as *σ*_*s*_, and *σ*_*n*_ are the normal stresses. Compressive stresses are positive by convention. The vertical stress is indicated by *σ*_*v*_, *P*_*i*_ represents a hypothetical mean stress (pressure) in the weak body and *P*_*s*_ represents the mean stress (pressure) in the surrounding region. For a general 3d state of stress the mean stress does not have to be exactly in the center of the corresponding Mohr circle. In the limit of negligible differential stress in the weak body, as in this case, all the normal stress components in the weak body will be equal to its pressure.

In general, the pressure in the fluid at the inclusion-matrix interface must be equal to the local normal traction in the matrix. If the matrix is stressed, then the pressure in the weak body must vary in order to be in force balance with the matrix. For the given parameters that are used here (Table 1) the pressure of the weak inclusion can be as high as ~3.3 times the lithostatic load (see Supplementary Information for derivation). Although we have considered an endmember scenario where we used a fluid-filled body as a weak analogue, relatively weak bodies such as weak lithologies and magma chambers under stress would have the same general behavior and could develop significant pressure variations from the lithostatic. This behavior has been suggested from grain to lithosphere scale^24–31^. The reason for the development of pressure variation in weak bodies is shown schematically in Fig. 3 and is explained further below.

**Table 1:** Typical values for the parameters used for scaling in models and in nature (Earth’s upper crust).

	${\boldsymbol{g}}\,[\frac{{\boldsymbol{m}}}{{{\boldsymbol{s}}}^{{\bf{2}}}}]$	${{\boldsymbol{\rho }}}_{{\boldsymbol{s}}}\,[\frac{{\boldsymbol{kg}}}{{{\boldsymbol{m}}}^{{\bf{3}}}}]$	${\boldsymbol{L}}\,[{\boldsymbol{m}}]$	${\boldsymbol{v}}\,[\frac{{\boldsymbol{m}}}{{\boldsymbol{s}}}]$	${\boldsymbol{C}}\,[{\boldsymbol{Pa}}]$	${\boldsymbol{\phi }}\,[{\boldsymbol{^\circ }}]$	$\frac{{\boldsymbol{C}}}{{\boldsymbol{\rho }}\mathrm{gL}}$
Model A	9.81	1,500	$6.7\cdot {10}^{-2}$	$2.8\cdot {10}^{-5}$	50	31	0.0507
Model B	9.81	1,500	$5.7\cdot {10}^{-2}$	$2.8\cdot {10}^{-5}$	50	31	0.0591
Nature	9.81	2,700	$15,000$	$3.1\cdot {10}^{-10}$	$20\cdot {10}^{6}$	30–35	0.0503

The characteristic velocities that are given are representative for tectonic processes that operate in nature and in the laboratory. Typical parameters for density, cohesion and angle of internal friction in natural rocks are taken from Burov (2015)^54^. Parameters for quartz sand are taken from Calignano *et al*.^55^. The previous authors^55^ report cohesion for quartz sand that was used between 30 and 70 Pa, here we use 50 Pa as an average value.

The development of stress variations can be better visualized with the utilization of Mohr-circle diagrams. Following the argumentation of Moulas *et al*. (2014, 2018)^26,32^, the stress conditions across a material interface that separates two rocks of different strength can be represented by two Mohr circles (Fig. 3b). The two circles have to have a common point as required by stress (traction) balance across the matrix-inclusion interface. The radii of the circles are proportional to the differential stress supported by the materials. A large Mohr circle would then be the one representative for the stronger lithology (matrix) while the small Mohr circle would be representative for the weak lithology (inclusion). The contact of two Mohr circles of different radii requires that the circles would not be concentric and most probably they will experience different mean-stress (pressure) values. If a weak body cannot support significant differential stress, its pressure could be close to one of the principal stresses of the strong surrounding body^29^ and its value would also depend on the local stress rotation. The magnitude of the stress and pressure variations does not depend on the absolute strength of the weak bodies but it rather correlates with the maximum differential stress in the model (e.g. Figure 3b). Although we used material (dry sand) with Mohr-Coulomb behaviour, the development of stress and pressure variations is not limited to plastic rheology^27–31^. Analytical solutions of viscous/elastic inclusions embedded in a matrix suggest that weak elliptical inclusions will develop stress variations in the presence of tectonic stress as a result of force balance^32,33^. More recent complex thermomechanical models suggest that weak viscous shear zones or boudins can develop significant stress and pressure variations upon deformation^28,30^.

The observation that the hydrostatic pressure practically did not relax after deformation ceased (Fig. 1) suggests that the stress level in the brittle layer remained approximately constant. This is actually an expected behavior for a non-creeping, time-insensitive rheology such as the one that is used in this work. Previous studies utilizing analogue experiments with sand have suggested that stresses drop once shear bands (thrust faults) are forming in the models which are in critical state^34,35^. However, we note that the scaling that was applied in this work does not allow the resolution of short-lived stress drops since this would also require the consideration of the inertial terms (equation 4; METHODS section). We therefore limit the applicability of our results to the long-term strength of the brittle crust.

Our results have certain implications when larger spatial and temporal scales are considered. Co-seismic faulting is sometimes expected to completely relax the stress of the upper crust as indicated by earthquake stress drops^36^. If that were the case in our experiments, stresses would have to relax significantly after every shear band formation and this behavior was not observed. In contrast the steady increase of the inclusion pressure was observed which is consistent with the general increase of differential stress in the model. This result is consistent with direct stress measurements from deep boreholes which indicate that the upper crust can be in a brittle-failure equilibrium even at stable intraplate areas^37–39^.

From a theoretical point of view, a complete stress drop during faulting and earthquake generation is inconsistent with the presence of mountains and crustal roots^40–42^. Mechanical models suggest that even without far-field compression, spatial variations in gravity potential energy (e.g. caused by topographic/density variations) require the preservation of stress and pressure variations as required by the conservation of linear momentum^43–47^. We therefore suggest that crustal stress and pressure variations like the ones investigated in this work can be maintained at geological timescales even after the far-field deformation has ceased.

## Conclusions

We have experimentally confirmed that a stressed heterogeneous crust will develop significant stress and pressure variations once deformed.

1. These variations are larger when weak bodies occur in a strong crust and they are the natural outcome of the conservation of linear momentum.
2. This behavior is not limited to a brittle crust and to plastic rheology as suggested by various mechanical studies.
3. The amplitude of the stress variations is proportional to the maximum differential stress in the model and therefore it is dominated by the strongest part of the system.
4. In compressive tectonic regimes and for standard material parameters the stress components of a weak body such a magma chamber can be >2 times larger than the lithostatic stress of the surrounding rocks.

These stress and pressure variations are expected to be preserved over geological timescales as required by the stability of mountain belts and crustal roots.

## Methods

### Experimental configuration

**Figure 4:**
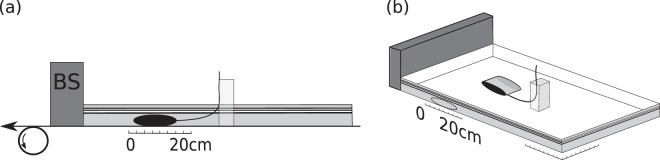
Initial model configuration. Side (**a**) and general (**b**) view of the experimental configuration. The figures are to scale. The backstop is indicated with “BS” in (**a**). The transparent grey color indicates the position of the metallic bar that is used to hold the upper part of the rubber tube straight. The sketch follows the scaling of Model A. Apart from the initial thickness, the rest of the Model parameters in A and B are the same.

We performed two experiments (Model A, B). For brittle layer we used dry quartz sand with a grain size of 100-300 μm and a density of 1500 kg·m^−3^ on a moving plastic sheet in a “conveyor-belt” configuration^9^. At the end of the layer, a rigid plate was placed as a backstop (Fig. 4a). Metallic bars laterally confined the layer. To create a low-strength perturbation we used a water-filled bladder connected with a thin tube that is open to the surface (Fig. 4). The initial thickness of the sand layer was 6.7 cm in Model A and 5.7 cm in Model B.

**Figure 5:**
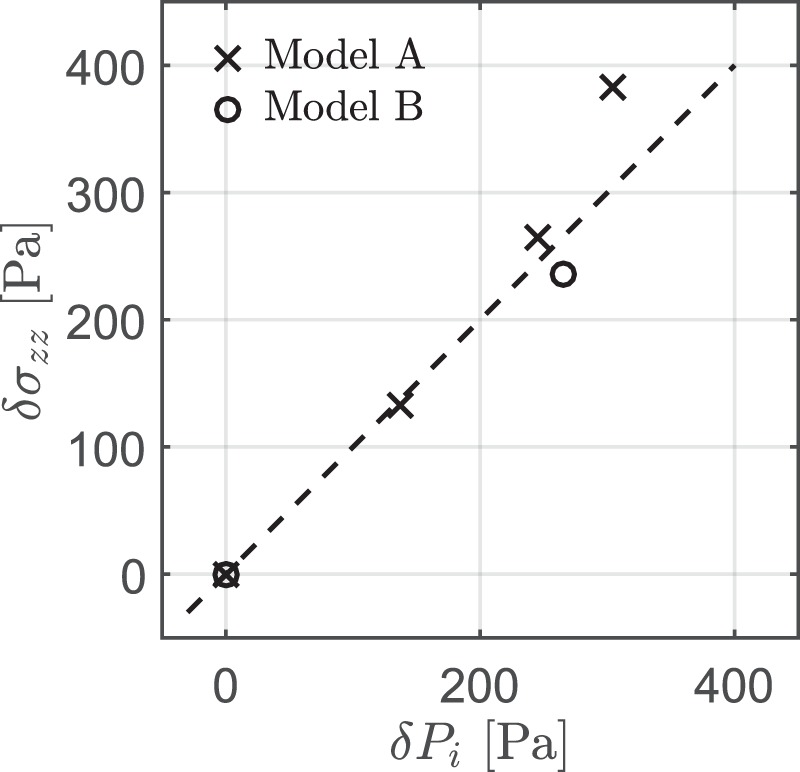
Calibration curve for the relationship between the inclusion pressure (*P*_*i*_) and lithostatic load (*σ*_*zz*_) for the two different models. The initial hydrostatic pressure of the inclusion was monitored once the bladder was covered with sand. Adding more sand above the bladder lead to a pressure increase. Comparison between the calculated lithostatic stress difference (*δσ*_*zz*_) and the measured hydrostatic pressure difference (*δP*_*i*_) showed that the maximum uncertainty of pressure determination is in the order of 60 Pa. The dashed line indicates the 1-1 relationship for reference.

By low-strength perturbation, we mean that this part of the system is not able to support high differential stress. The bladder was only partially filled with water in order to make it more deformable. We monitored the pressure (*P*_*i*_) inside the low strength inclusion by recording the hydrostatic head on the thin rubber tube that is open to the surface (opening diameter: 0.6 cm). The rubber tube is elastic and deformable which allows the inclusion to deform. A small metallic bar is placed in a distance 14.5 cm from the inclusion in order to hold the rubber tube in a vertical position at the surface. The experiments were stopped at a relative small strain and therefore the metallic object did not interact or came closer to the inclusion. The hydrostatic pressure of water in the inclusion was measured and its accuracy has been checked in various tests as well as before the experiment during the burial of the inclusion (Fig. 5). Different hydrostatic-pressure measurements were obtained after the inclusion was completely covered and the difference of the pressure head (*δP*_*i*_) was compared to the difference of the lithostatic load (*δσ*_*zz*_) that was used until the final thickness of the sand was reached.

### Scaling

We consider the conservation of linear momentum^48,49^ in order to determine the scaling relations in our experiments:

4 $$\frac{\partial {{\sigma }}_{{ij}}}{\partial {{x}}_{{j}}}+{\rho }{{g}}_{{i}}={\rho }\frac{{D}{{v}}_{{i}}}{{Dt}}$$

where *i* is a free index (*i* = 1, 2, 3), *x* is the Cartesian coordinate, *σ*_*ij*_ are the components of the stress tensor and *D*/*Dt* is the Lagrangian (material) derivative. Einstein summation rules on repeated indices are applied. The acceleration of gravity in the *i*^th^ direction is *g*_*i*_, velocity in the same direction is *v*_*i*_, and *ρ* represents the density of the material. The relation between normal *σ*_*n*_ and shear stress *σ*_*s*_ for a Mohr-Coulomb failure criterion is^50^:

5 $${{\sigma }}_{{s}}=\,{\tan }({\phi }){{\sigma }}_{{n}}+{C}$$

where *φ* is the angle of internal friction and *C* is the rock cohesion. We chose to scale the previous equations by using the initial layer thickness (*L*), sand density (*ρ*_*s*_), the magnitude of gravity acceleration (*g*) and velocity (*v*) as independent scales. The characteristic stress used for nondimensionalization is chosen to be the initial lithostatic stress and is given by:

6 $${{\sigma }}_{{zz}}^{{i}}={{\rho }}_{{s}}{gL}$$

This choice of scaling is useful for models where gravity is important^3^. Equations (4) and (5) can thus be written as shown below (tildes denote non-dimensional variables).

7 $$\frac{\partial {\tilde{{\sigma }}}_{{\boldsymbol{i}}{j}}}{\partial {\tilde{{x}}}_{{j}}}+\tilde{{{\rho }}_{{s}}}=\tilde{{{\rho }}_{{s}}}\frac{{D}{\tilde{{v}}}_{{i}}}{D\tilde{{t}}}(\frac{{{v}}^{2}}{{gL}})$$

8 $${\tilde{{\sigma }}}_{{s}}=\,{\tan }({\phi }){\tilde{{\sigma }}}_{{n}}+(\frac{{C}}{{{\rho }}_{{s}}{gL}})$$

The terms in the brackets in equations (7) and (8) are non-dimensional numbers that have to be similar in nature and experiments in order to have dynamic (equation 7) and rheological (equation 8) similarity^51,52^. The non-dimensional term in brackets in equation (7) is related to inertial forces. This number is much smaller than unity $(\frac{{v}^{2}}{gL}\ll 1)$ for typical tectonic rates in nature and in the laboratory and can safely be neglected. The ratio of cohesion to lithostatic stress $(\frac{C}{{\rho }_{s}gL})$ determines the significance of rock cohesion in systems where lithostatic stresses are significant. This ratio is similar in nature and in the analogue experiments as shown in Table 1. An additional non-dimensional parameter that we have to consider is the angle of internal friction (*φ*) in equation (8). Direct stress measurements, experimental measurements and laboratory studies suggest that this number is similar in nature and in experiment (Table 1) and its value is approximately 30–35°^37–39,53^.

## Supplementary information

Supplementary Information

## Data Availability

All the data are available upon request to E.M.
