# Supplementary material for: Pressure build-up and stress variations within the Earth’s crust in the light of analogue models
Source: Sci Rep. 2019 Feb 19;9:2310. doi: 10.1038/s41598-018-38256-1 (PMC6381219; doi:10.1038/s41598-018-38256-1)
Supplement: Supplementary file 1 — Supplementary Information [file 41598_2018_38256_MOESM1_ESM.docx]

***Supplementary Information***

***Pressure build-up and stress variations within the Earth’s crust in the light of analogue models***

 Evangelos Moulas^1*^, Dimitrios Sokoutis^2,3^ & Ernst Willingshofer^2^

(^*^corresponding author)

^1^Institute of Earth Sciences, University of Lausanne, Switzerland (evangelos.moulas@unil.ch)

^2^Department of Earth Sciences, Utrecht University, Netherlands

^3^University of Oslo, Department of Geosciences, PO Box 1047 Blindern, NO-316 Oslo, Norway

This document contains the analytical derivation for the potential pressure magnitude in a weak inclusion under stress.

**Analytical Derivation**

In order to estimate the potential magnitude of the pressure of a weak body in a stressed crust, we will consider a crustal region in plane-strain shortening. For simplicity, we will assume that the crust is in plastic state that is described by the Mohr-Coulomb failure criterion (equation 5 in the main text). To a first approximation we can take the maximum normal stress to be horizontal ($\sigma_{1}$) and ($\sigma_{3}$) as the vertical lithostatic stress $\sigma_{zz}=\rho_{s}gh$ (compressive stresses are taken as positive). The off-plane normal stress ($\sigma_{2}$) can be approximated to be:

$\sigma_{2}=\frac{\sigma_{1}+\sigma_{3}}{2}$ (S1)

The Mohr-Coulomb criterion can be expressed in terms of principal stresses as^1–3^:

$\sin\left( \varphi\right)\left( \frac{\sigma_{1}+\sigma_{3}}{2}+\frac{C}{\tan\left( \varphi\right)} \right)=\frac{\sigma_{1}-\sigma_{3}}{2}$ (S2)

By considering also (S1), pressure (mean stress) is given by:

$P=\frac{\sigma_{1}+\sigma_{2}+\sigma_{3}}{3}=\frac{\sigma_{1}+\sigma_{3}}{2}$ (S3)

Solving (S2) with respect to $\sigma_{1}$ leads to the following relation.

$\sigma_{1}=\frac{\sigma_{3}\left( \sin\left( \varphi\right)+1 \right)+2 C cos(\varphi)}{1-sin(\varphi)}$ (S4)

Replacing the previous expression in (S3), substituting the lithostatic stress for $\sigma_{3}$, and solving for $P$ yields:

$P=\frac{\rho_{s}gh+C cos(\varphi)}{1-sin(\varphi)}$ (S5)

If cohesion is neglected and $\varphi$ is taken to be 30°, (S5) reduces to the classic formula^2^:

$P=2\rho_{s}gh$ (S6)

Substitution for the values of cohesion and internal friction angle from Model A into (S4) and simplifying leads to

$\frac{\sigma_{1}}{\rho_{s}gh}=3.3$ (S7)

Equation (S7) shows that the maximum normal stress that can develop in a brittle crust in plastic state can be as high as 3.3 times the lithostatic stress.

**References**

1. Jaeger, J. C., Cook, N. G. W. & Zimmerman, R. W. *Fundamentals of Rock Mechanics*. (Blackwell Publishing, 2007).

2. Petrini, K. & Podladchikov, Y. Lithospheric pressure–depth relationship in compressive regions of thickened crust. *Journal of Metamorphic Geology* **18**, 67–77 (2000).

3. Yamato, P. & Brun, J. P. Metamorphic record of catastrophic pressure drops in subduction zones. *Nature Geosci* **10**, 46–50 (2017).
